# DDX23-Linc00630-HDAC1 axis activates the Notch pathway to promote metastasis

**DOI:** 10.18632/oncotarget.17156

**Published:** 2017-04-17

**Authors:** Guozhang Mao, Hui Jin, Liuguang Wu

**Affiliations:** ^1^ Department of Cardio-Thoracic Surgery, Zhoukou Center Hospital of Henan Province, Henan 466000, China

**Keywords:** Linc00630, HDAC1, DDX23, Notch signaling pathway, NSCLC

## Abstract

Emerging studies demonstrated the roles of long non-coding RNAs (LncRNAs) are being implicated in the progression of many cancers. Here we report the discovery of a critical role for the linc00630 in the development of Non-Small-Cell Lung Cancers (NSCLCs). Screening from the microarray of six paired NSCLCs and adjacent non-tumor tissues, linc00630 showed a significantly higher RNA levels in NSCLCs. With the higher level confirmed in a separate cohort 90 NSCLCs patients, overexpressed of linc00630 also positive associated with tumor size, TNM tumor stage, lymph node status positive and overall patient outcomes. Linc00630 overexpression increased cell proliferation and metastasis *in vitro* and *in vivo* whereas linc00630 silencing had opposite effects. By RNA pull-down and mass spectrometry we identified Histone deacetylases 1 (HDAC1) and DEAD-box helicase 23 (DDX23) as the linc00630-binding protein that associated with mechanism of linc00630. DDX23 can specific bind with the promoter of Linc00630 to up-regulate the RNA level and high level of linc00630 strength the protein stability of HDAC1 to regulate the downstream pathway.

Our study demonstrates the effectiveness of Linc00630 oligonucleotide-based promotion of NSCLCs metastasis and proliferation, illuminating a new basis of DDX23-Linc00630-HDAC1 signal axis for understanding its pathogenicity, which could be further developed as a valuable therapeutic strategy.

## INTRODUCTION

Lung cancer is the leading cause of cancer-related death worldwide. Among all lung cancer cases, non-small-cell lung cancers (NSCLCs) account for approximately 85% [1, 2], which are at locally advanced or metastatic stage at diagnosis [3–5]. Although the traditional therapeutic strategies have been tremendously improved, such as tyrosine kinase inhibitors (TKIs) of the epidermal growth factor receptor (EGFR) and immune checkpoint inhibitors [6–8], have been successfully used in clinical practice [9], but the five-year overall survival of lung cancer of all stages combined remains as low as 15% [10]. Such unfavorable outcome could be at least partially attributed to the poor understanding of the pathogenesis of NSCLC, as well as lack of early diagnostic biomarkers and therapeutic targets. Though alterations in oncogenes and tumor-suppressive genes have been reported in NSCLC [11–13], the precise molecular mechanisms underlying NSCLC pathogenesis still remain to be further elaborated. Hence, better understanding of the oncogenesis is critical for the advance of diagnostic markers and aid novel effective therapies for NSCLC patients [14].

With the development of technological approaches, such as lncRNA microarray and RNA sequencing, more and more lncRNAs have been found to be dysregulated in cancer, which function as oncogenes or tumor suppressors [15–18]. Long non-coding RNAs (lncRNAs) are a class of non-coding RNAs whose length is more than 200 nucleotides (nt) in length without protein-coding capacity. LncRNAs are increasingly recognized to play major regulatory roles in diverse biological processes and diseases by regulating gene expression at the chromatin organization, transcriptional and post-transcriptional levels [19–22]. Some of these lncRNAs are associated with different stages of NSCLCs, some are specifically overexpressed in one of the lung cancer subtypes, and some are involved in drug resistance [23, 24]. These findings suggest the important roles of lncRNAs in the pathogenesis and treatment of NSCLCs. However, only a small number of lncRNAs have been well characterized, whereas functions of most lncRNAs remain to be elucidated.

In the present study, through microarray analysis of NSCLCs tissues, we found a number of lncRNAs dysregulated in NSCLCs compared with paired nontumoral tissues. Among them, we further characterized the clinicopathologic relevance of a novel lincRNA linc00630 in NSCLCs progression which showed the significant upregulation. Linc00630 expression was positively correlated with TNM stage, tumor size and was negatively correlated with metastasis and overall survival (OS) time. Linc00630 can interact with HDAC1 protein and DDX23 transcription factor to promoting proliferative and invasion functions of NSCLCs cells. We provided *in vitro* and *in vivo* data to demonstrate that linc00630, which is a target of DDX23, increased cell proliferation and invasion of NSCLCs by stabilization of HDAC1 protein.

## RESULTS

### Linc00630 significantly up-regulated in NSCLC

**Figure 1:**
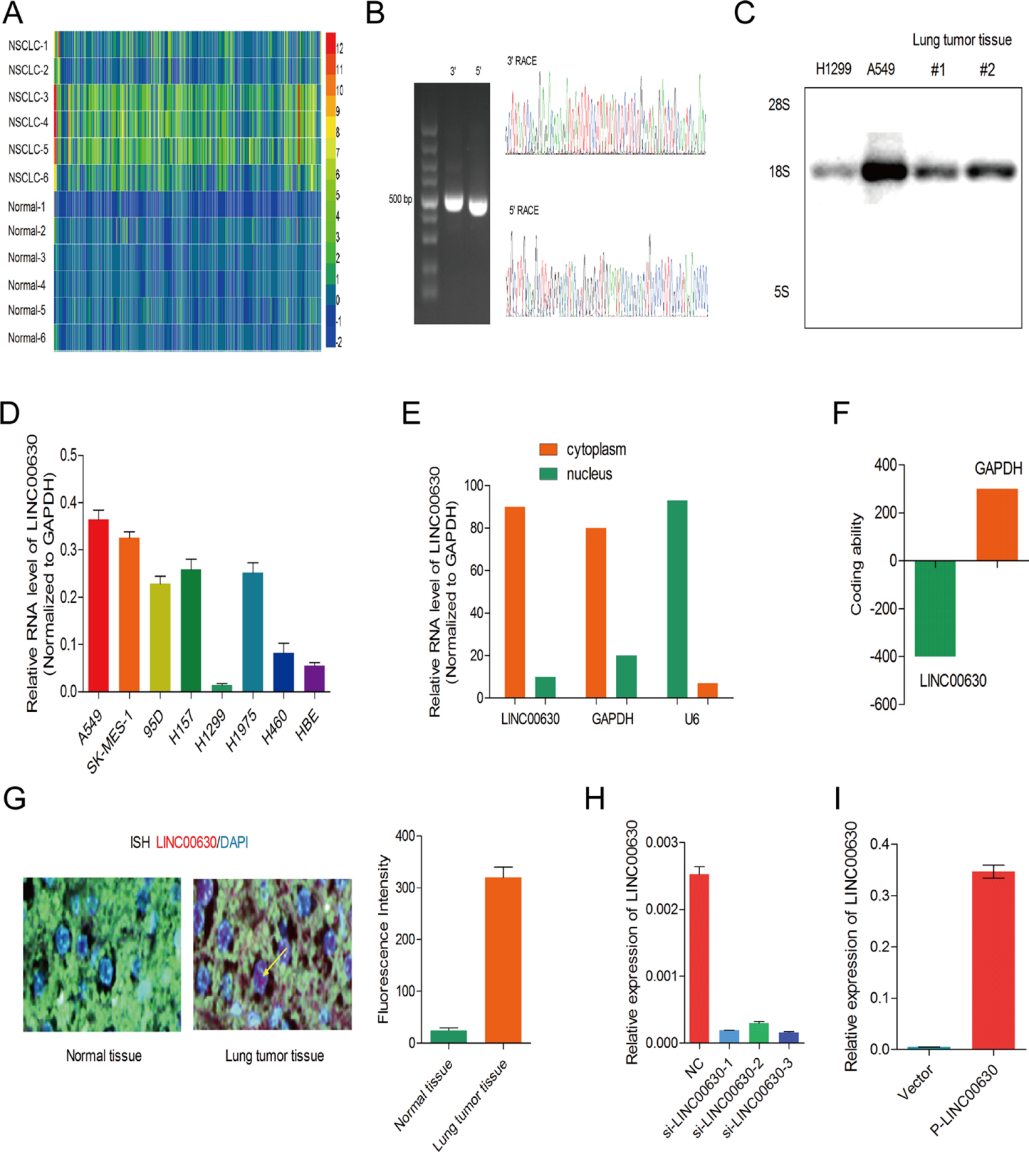
Screened Linc00630 significantly up-regulated in NSCLC from the microarray (**A**) Hierarchical clustering analysis of the top 470 lncRNAs that were differentially expressed (> 2-fold; *p* < 0.05) between NSCLC samples (tumor) and paired non-tumor samples (NT, non-tumor). (**B**) 5′ RACE and 3′ RACE to identify the full-length of linc00630 in A549 cells. Left, representative images and the boundary of the PCR products from. Right, the nucleotide sequence of full-length human linc00630 in A549 cells. (**C**) Northern blot for detected the full-length of linc00630, incubation of total RNA from A549. (**D**) linc00630 RNA levels of in NSCLC cell lines. β-actin served as the control. Values are expressed as mean ± SEM, *n* = 3. (**E**) Relative subcellular distribution of linc00630 in NSCLC cells, determined by qPCR. U6 served as the nuclear internal control, β-actin served as cytoplasmic internal control. (**F**) PhyloCSF software prediction for the protein-coding potential of linc00630. (**G**) ISH assay to determine the subcellular location of linc00630 in NSCLC tissues. (**H**) The efficiency of three independent siRNA for linc00630 silencing. (**I**) The fold change of overexpressed linc00630 by eukaryotic expression vector. Values are expressed as mean ± SEM, *n* = 3.

The Arraystar Human LncRNA microarray V2.0 was used to profiling the expression level of LncRNA in six paired NSCLC tissues and paired adjacent normal tissues (Figure 1A). In total, 230 significantly deregulated LncRNAs were collected and analyized by the databases such NCBI (National center of biotechnology information), UCSC (Univeraity of California santa cruz) and Ensembl. To selected the potential oncogentic LncRNAs in NSCLC tumorigenesis, we analysis the RNA levels in 90 paired NSCLC tissues, finally, we choose linc00630, which the expression was most significantly upregulation, for further investigation.

We first identified the full poly (A)-positive sequence of linc00630 through rapid amplification of cDNA ends (RACE) (Figure 1B) and performed Northern-blot analysis to confirm the full-length of Linc00630 RNA size 2117 bp (Figure 1C). Linc00630 was expressed extensively in NSCLC cell lines, we performed qRT-PCR analysis to determine the expression level of linc00630 in 8 human NSCLC cell lines which include both squamous carcinoma and adenocarcinoma. It was determined that linc00630 expression was elevated to in 6 lung cancer cell lines, whereas linc00630expression was lower in H1299 and higher in A549 than that in human bronchial epithelial cells (HBEs) (Figure 1D). We also have separated the nuclear and cytoplasm fractions of A549 cells and performed real-time PCR. We found that linc00630 was mainly located in the cytoplasm (Figure 1E), which implies that linc00630 may exert both transcription and post-transcriptional level regulatory functions in NSCLC cell lines. both transcription and post-transcriptional level regulatory functions in NSCLC cell lines. In addition, phyloCSF software, was used to calculate its protein coding potential, and a score was assigned based on its sequence, and the score was less than 800 was considered as the noncoding RNA [25]. The phyloCSF score for linc00630 was −390, indicating that linc00630 has no protein-coding potential (Figure 1F). Analyzed by *In situ* hybridization assay also confirmed, linc00630 has a high-expression in the cytoplasm of NSCLC tissues(Figure 1G). To investigate the biogical effect of linc00630 of NSCLC cells, we used three specific siRNAs to knock down endogenous linc00630 in A549 and developed a linc00630 overexpression plasmid using transfection of pcDNA3.1-linc00630 in H1299 to establish the cell model (Figure 1H–1I).

To sum up, linc00630 is a novel long non-coding RNA which has high expression in NSCLC cell lines and tissues.

### Clinically relevant of linc00673 predicts poor prognosis of NSCLC patients

**Figure 2:**
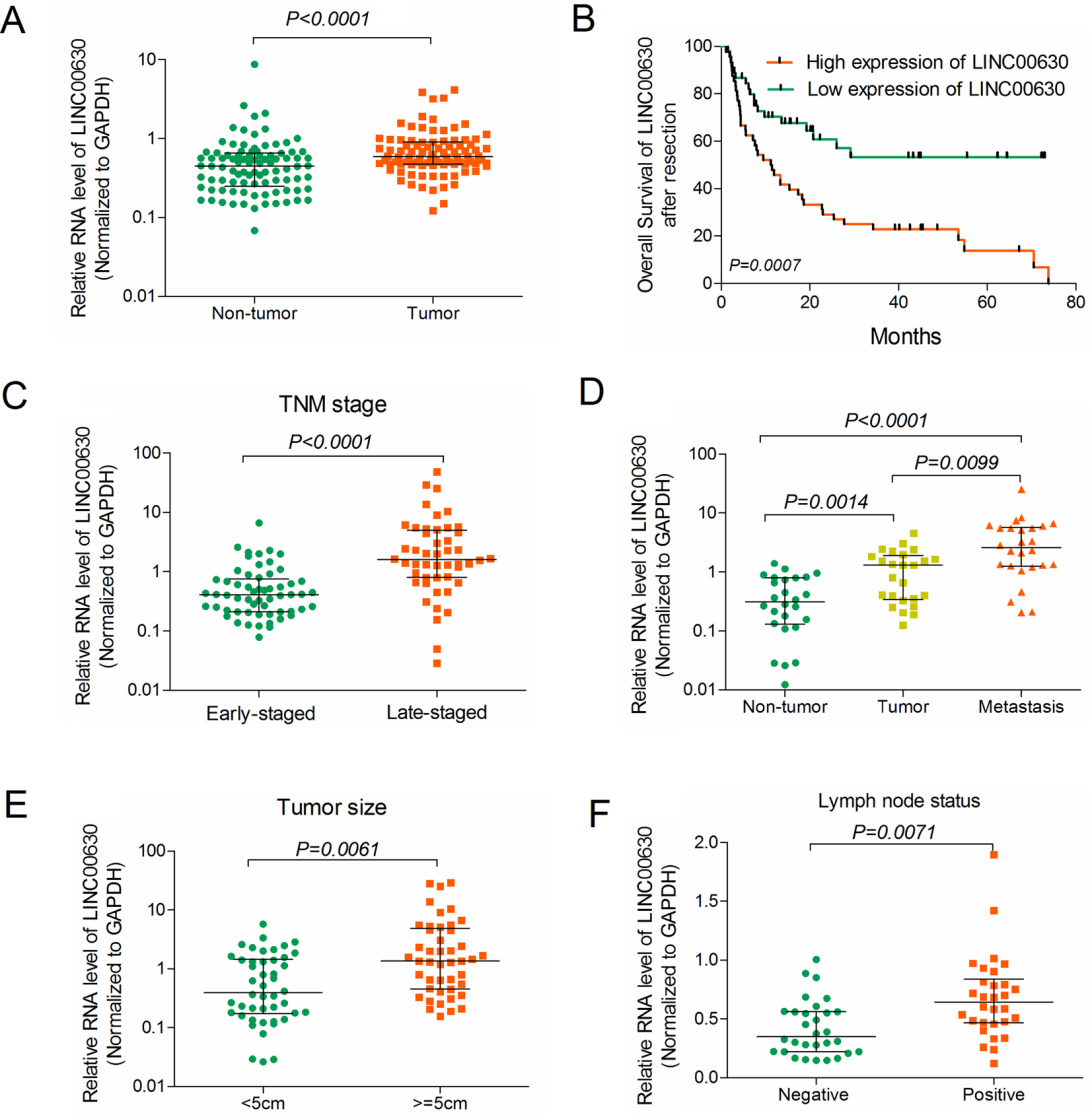
Clinically relevant of linc00673 predicts poor prognosis in NSCLC patients (**A**) Linc00630 expression was analyzed by qRT-PCR in NSCLC samples and adjacent non-tumor liver tissues (cohort 1, *n* = 90). β-actin served as the control. (**B**) Kaplan-Meier survival analysis of overall survival (log-rank test) in 90 paired NSCLC patients. (**C**–**F**) Relative linc00630 RNA expression in 90 NSCLC tumor samples, with TNM-stage (Early or Late) (C); with or without Metastasis (D); with Tumor size (< 5 cm or > 5 cm); with lymph node status (Negative or Positive) Values are expressed as median with interquartile range, *n* = 3.

In order to ascertain linc00630 was differentially expressed in the NSCLC tissues, we analysis the RNA level of linc00630 in 90 paired clinical NSCLC tissues and adjacent normal tissues by qRT-PCR. Results showed that linc00630 was up-regulated in clinical NSCLC tissues (*P* < 0.0001, Figure 2A). The Kaplan-Meier analyses showed that high level of linc00630 have predictive worse clinical outcomes of NSCLC patients (Figure 2B). Besides, We also evaluated the correlation of linc00630 expression with patients' clinical-pathological parameters (i.e., maximum diameter, lymphatic metastasis or TNM stage) to assess its clinical significance. Our results showed that larger tumors, with lymph node metastasis, or more advanced tumors, had higher linc00630 expression levels (Figure 2C–2F). Nevertheless, there was no significant relationship between linc00630 expression and other clinical characteristics, such as age, gender, differentiation and smoking history (*P* > 0.05, Supplementary Table 1). These data demonstrate that linc00630 might be exert as an oncogene in the progression of NSCLC and may potentially serve as diagnostic biomarker for NSCLC patients.

### Linc00630 promotes the migration, invasion and proliferation of NSCLCs cells *in vitro* and *in vivo*

**Figure 3:**
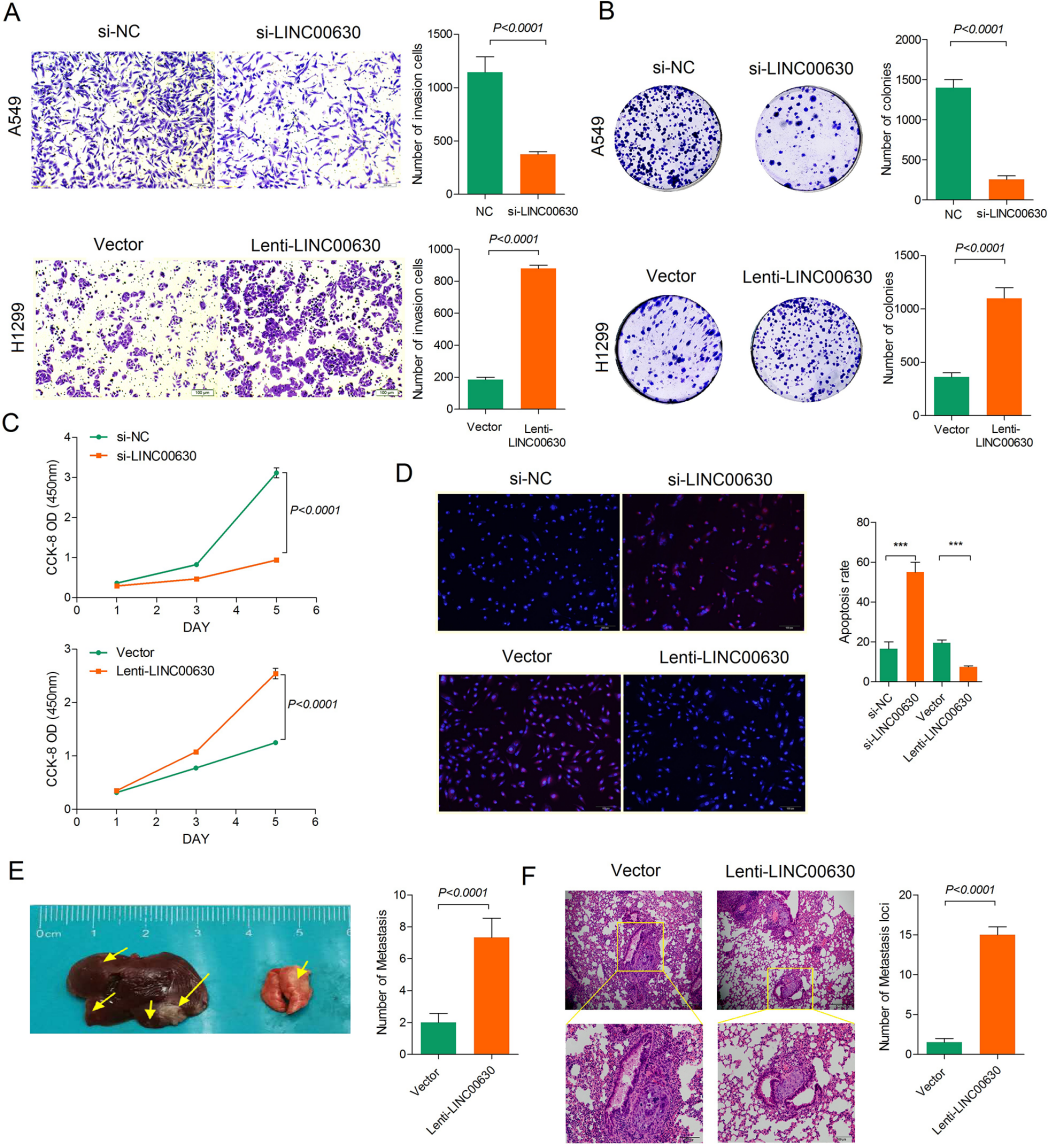
Linc00630 promotes the migration, invasion and proliferation of NSCLCs cells *in vitro* and *in vivo* (**A**) Transwell invasion assays for A549 and H1299 cells infected with the lentivirus expressing linc00630 or the control. (**B**) Colony formation assays for A549 and H1299 cells infected with the lentivirus expressing linc00630 or the control. (**C**) The cell growth rates were determined with CCK-8 proliferation assays. (**D**) EDU (red)/DAPI (blue) immunostaining assay were determined the cell apoptosis. (**E**) *In vivo* metastatic events in A549 cells infected with the lentivirus expressing linc00630 or the control. The cells were orthotopically injected into the lungs of nude mice. 50 days later, the mice were sacrificed, and the lungs were subjected to immunohistochemical staining. (**F**) Hematoxylin and eosin (H&E) staining of the sections with metastatic nodules in the lung. The numbers of metastatic nodules were counted and analyzed using Student' *t* test. Values are expressed as mean ± SEM, *n* = 3.

Linc00630 expression was higher in metastatic tumor tissues than in normal tissues, suggesting its potential role in tumor metastasis. To investigate the effect of linc00630 on NSCLC cell migration and invasion, we used two specific siRNAs to knock down endogenous linc00630 in A549 and developed a linc00630 overexpression plasmid using transfection of pcDNA3.1-linc00630 in H1299. Transwell assays were performed following transfection with si-linc00630 or p-linc00630. Reduced cell migration and invasion were observed in linc00630-depleted NSCLC cells as compared to the control cells (Figure 3A). Next, Colony formation assay showed that knockdown the expression of linc00630 greatly attenuated the colony-forming ability of A549, while an increased of linc00630 expression enhanced colony-formation ability of H1299 (Figure 3B). In addition, CCK-8 assays were used to determine cell viability in the NSCLC cell lines. siRNA transfection-mediated linc00630 knockdown resulted in a significant decrease in cell viability rate in A549, H1299 cells displayed a higher cell viability rate after linc00630 overexpression relative to negative control (Figure 3C). These observations were further confirmed by EDU (red)/DAPI (blue) immunostaining assay (Figure 3D). Collectively, these results validated a positive role of linc00630 in promoting NSCLC cell proliferation, migration and invasion *in vitro*.

To further analysis the oncogenic role of linc00630, we established metastatic model by caudal vein *in vivo*. H1299 cells stably transfected with overexpression of linc00630 or an empty vector were injected into male nude mice. Seven weeks after post-injection, linc00630 overexpression group dramatically increased tumor metastasis, which was determined by the metastasis loci numbers in Hemotoxylin and Eosin staining (H&E) staining, relative to the control group (Figure 3E and 3F). Taken together, we highlighted an important role of linc00630 in human NSCLC, however, the mechanism governing the oncogenic role of linc00673 in such this disease have yet to be elucidated.

### Linc00630 associates with HDAC1 and DDX23 in NSCLC cells

**Figure 4:**
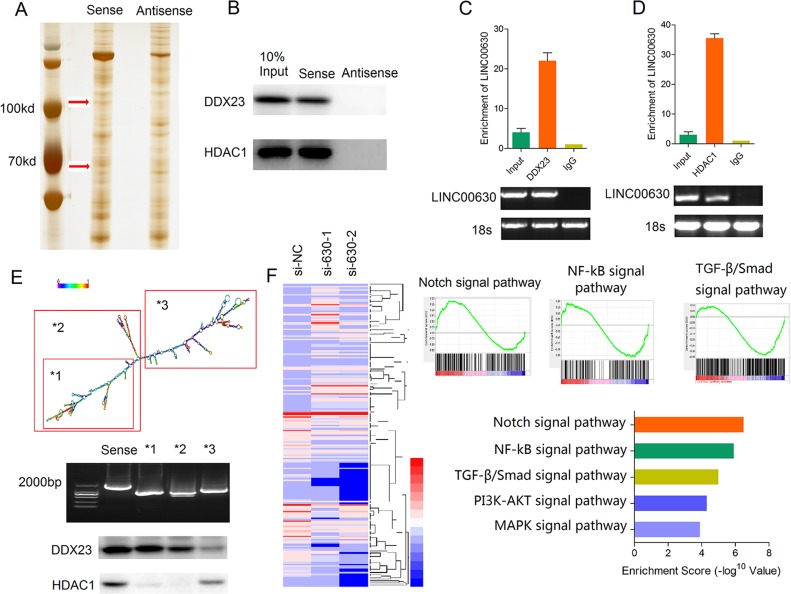
Linc00630 associates with HDAC1 and DDX23 in NSCLC cells (**A**) Silver stained SDS-PAGE gel of proteins pulldown from A549 cell extract by linc00630 and its antisense RNA. The arrow indicates the region of the gel excised for mass spectrometry. (**B**) Western blot analysis detected the specific association of HDAC1 and DDX23 with linc00630 (*n* = 3). (**C**, **D**) RIP experiments were performed using the HDAC1 antibody (C) and DDX23 antibody (D) for immunoprecipitation (IP) and a primer to detect linc00630. RIP enrichment was determined relative to the input controls (*n* = 3). (**E**) Western-blot to detect the binding site of HDAC1 and DDX23 with linc00630 by deletion mapping of the linc00630 in A549 cells. (**F**) Gene expression profiles from A549 cells transfected with the linc00630 siRNAs or the control. Gene set enrichment analyses used to identify the differential gene profiles, between A549 cells transfected with the linc00630 siRNAs and the control. Values are expressed as mean ± SEM, *n* = 3.

Recent studies have suggested that lncRNAs participate in molecular regulation pathways through interacting with protein partners. Thus, we firstly analysis the binding proteins of linc00630 in A549 NSCLC cells by RNA-pulldown assays. Biotin-labeled linc00630 were incubation with A549 total protein lysis. RNA-associated proteins were analyzed by SDS/PAGE and silver staining (Figure 4A). Three distinct bands specific to linc00630 were excised and subjected to mass spectrometry. HDAC1 and DDX23 was detected by western blotting from three independent RNA pull-down assays in cell extracts from A549 cells (Figure 4B). The specificity of this interaction was further verified with RNA immunoprecipitation (Figure 4C and 4D). Notably, according to the RNA folding structure predicted by http://www.lncipedia.org/, we performed deletion-mapping analyses identified that 3′-end segment of linc00630 is required for the interaction with HDAC1, and DDX23 specific binding with the 5′ -end segment (Figure 4E). In order to analyze the linc00630-associated gene transcriptional changes, we applied RNA transcriptome sequencing to assess the gene expression profiles of linc00630-knockdown A549 cells and control cells. This unbiased genome-scale analysis identified 420 differentially expressed transcripts log_2_ (Fold Change) > 1 and *P* < 0.05 in NSCLC cells after linc00630 knockdown compared to controls, including 219 downregulated genes and 201 upregulated genes (Figure 4F). Furthermore, to investigate the functional processes or signaling pathways that affected by linc00630, Gene Set Enrichment Analysis (GSEA) analysis were performed. Notch signaling pathway was top1 involved in the affected functional processes in si-linc00630 cells.

Taken together, these results showed linc00630 could specifically binding with HDAC1 and DDX23, and linc00630 may regulated the NSCLC cells invasion and proliferation through the Notch signaling pathway. The mechanisms between them need further explored.

### Linc00630 stabilized HDAC1 and it's the direct target of DDX23 in NSCLC cells

**Figure 5:**
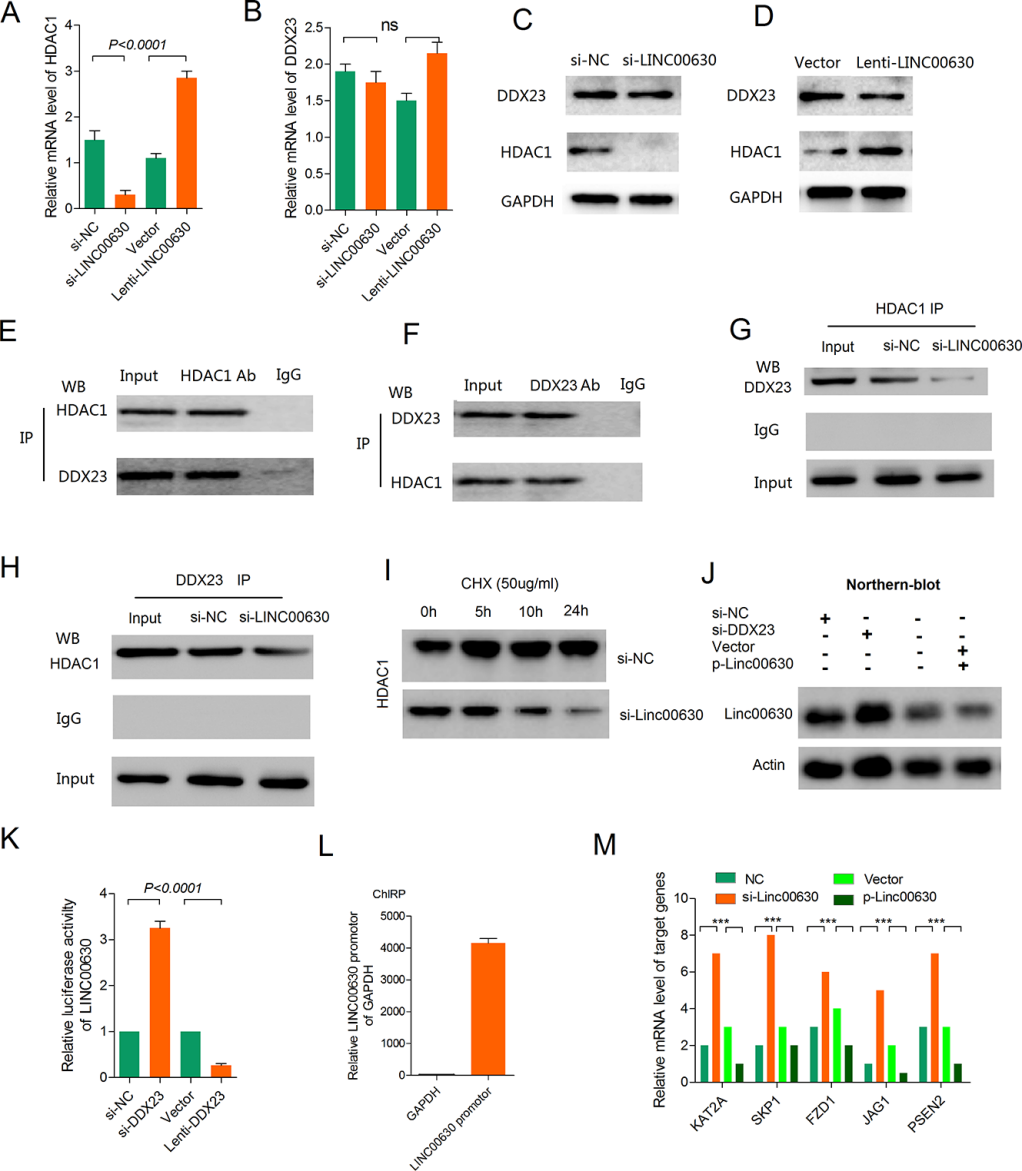
Linc00630 stabilized HDAC1 and it's the direct target of DDX23 in NSCLC cells (**A**–**B**) QPCR detected the mRNA level of HDAC1 (A) and DDX23 (B) when knockdown or overexpressing linc00630 in A549 cells. (**C**) Western blot to detect the protein level of HDAC1 and DDX23 when knockdown of linc00630 in A549 cells. (**D**) Western blot to detect the protein level of HDAC1 and DDX23 when overexpressing of linc00630 in A549 cells. (**E**–**F**) Co-immunoprecipitation to detecte whether HDAC1 could binding with DDX23 in A549 cells. (**G**–**H**) Knockdown of linc00630 could abolish the binding state of HDAC1 and DDX23. (**I**) Knockdown of linc00630 in A549 cells and control cells were incubated with the protein synthesis inhibitor cycloheximide (CHX, 0.5 μg/μl) for 24 hours. (**J**) Northern blot to detect the RNA level when knockdown or overexpressing of DDX23. (**K**) Luciferase assays for SMMC-7721 cells infected with the lentivirus expressing DDX23 or transfected with DDX23 siRNAs. (**L**) The enrichment of linc00630 promoter by ChIRP assay. (**M**) The key genes of Notch pathway were regulated by linc00630. Values are expressed as mean ± SEM, *n* = 3. Values are expressed as mean ± SEM, *n* = 3.

Next, we explore the mechanisms of these interaction. Firstly, we detected the mRNA level of HDAC1 and DDX23 when knockdown or overexpression of linc00630. We did not observe a significant change in HDAC1 and DDX23 mRNA levels (Figure 5A and 5B). And we also test the protein level of these two proteins, results showed a significant downregulation of the HDAC1 protein upon linc00630 knockdown, and upregulation of HDAC1 when overexpressing linc00630, but the protein level of DDX23 don't observe a significant change in the same treatment in A549 cells (Figure 5C and 5D). This phenomenon hints us to explore the role of linc00630 played in these two binding proteins. To this end, we performed Co-Immunoprecipitation (Co-IP) assay demonstrated HDAC1 can binding with DDX23 in A549 cells (Figure 5E and 5F). And knockdown of linc00630 could abolished the interaction between HDAC1 and DDX23 (Figure 5G and 5H). Based on this finding, we hypothesize that linc00630 binds to HDAC1 and affects its biological activity at the translational or post-translational level. To identify these hypotheses, we observed the expression of HDAC1 proteins in A549 cells incubated with the protein synthesis inhibitor cycloheximide (CHX). As shown in Figure 5I, knockdown of linc00630 accelerated the degradation in 24 hours treatment contrasted with control group. These results suggest that linc00630 might strengthen the stability of HDAC1 protein. Besides, in view of linc00630 don't affect the protein and mRNA level of DDX23, we wonder whether linc00630 regulated by DDX23 at transcription level. So we performed Northern-blot assay when knockdown or overexpression of DDX23, results showed overexpressed DDX23 could increase RNA level of linc00630, and knockdown DDX23 could significantly decreased the linc00630 RNA level (Figure 5J). This result hints us, whether DDX23 as a transcription factor which specific binding at the promoter of linc00630 to enhanced the transcriptional activity. And then, we cloned the promoter of linc00630 in luciferase report vector. Results showed overexpression DDX23 could increase the luciferase activity of linc00630 and knockdown DDX23 decrease the luciferase activity for further demonstrated DDX23 could enhance the transcription activity of linc00630 in A549 cells (Figure 5K). In order to verify the result, we performed the Chromatin Isolation by RNA Purification (ChIRP) assay, results in Figure 5L, results showed the fold enrichment of linc00630 promotor fragments in DDX23 ChIRP analysis with linc00630 sDNA as probe. These results illustrated DDX23 can specific binding with the promotor of linc00630 which enhance the transcription activity of linc00630.

GSEA revealed linc00630 related to Notch signaling pathway. The top-scoring genes recurring in the four gene sets included key cancer genes, KAT2A, SKP1, FZD1, JAG1 and PSEN2. Real-time PCR confirmed that alteration of linc00630 expression dramatically affected the key tumorigenesis gene signatures (Figure 5M).

Taken together, these data suggest that linc00630 may be an important modulator in NSCLC via stabilized HDAC1 and activated by DDX23 to activate the Notch signaling pathway.

### A positive correlation between HDAC1/ DDX23/ linc00630 in NSCLC tissues

**Figure 6:**
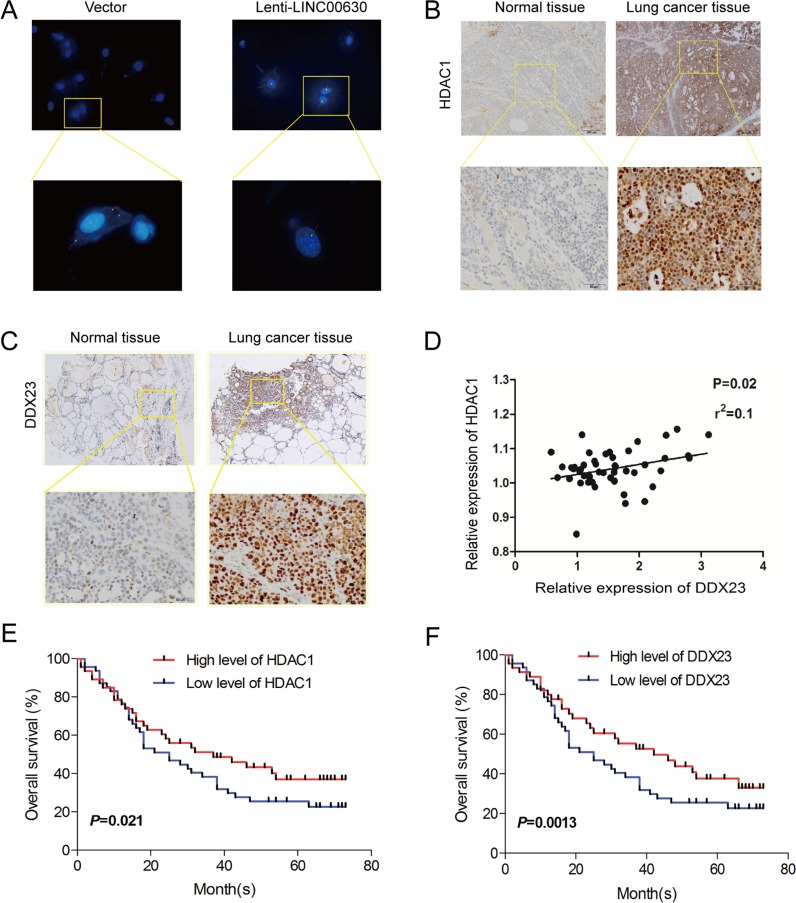
A positive correlation between HDAC1/ DDX23/ linc00630 in NSCLC tissues (**A**) RNAScope detection of linc00630 expression in NSCLC cells. (**B**, **C**) Immunohistochemical staining using antibodies against HDAC1 and DDX23. (**D**–**F**) Pearson's correlation analysis comparing staining density between linc00630 expression and HDAC1 and DDX23.

Based on the results which linc00630 were mainly located in cytoplasm, and it performed its mechanism in the nuclear. So we performed RNA scope assay to analysis the subcellular distribution when overexpressed endogenic linc00630, and we found high level of linc00630 striking enriched in the nuclear which support the mechanism binding with HDAC1 and regulated by DDX23 (Figure 6A). To determine the pathological significance of HDAC1 and DDX23 in NSCLC tissues, we analysis their levels via immune-histochemical (IHC) staining of 95 NSCLC and adjacent normal tissues samples. In this cohort, linc00630 showed a significantly higher expression level in NSCLC tissue, and HDAC1 and DDX23 showed they also have a high expression in NSCLC tissues (Figure 6B and 6C). At last, we detected the correlation between HDAC1/DDX23/linc00630, results showed HDAC1 and DDX23 had a positive correlation in NSCLC tissues (Figure 6D). Both HDAC1 and DDX23 could predicted the worse outcomes of NSCLC patients (Figure 6E, 6F and and Supplementary Figure 1). And we also demonstrated the DDX23 plays an oncogenic roles in NSCLC (Supplementary Figure 2). These results illustrated linc00630 may act as scaffold of HDAC1 and DDX23 to promoted the migration and invasion of NSCLC cells. And the flow aixs of DDX23-linc00630-HDAC1 were fluency, and the oncogenic aixs could be a potential target of NSCLC treatment.

## DISCUSSION

To date, a mounting number of protein-coding genes as valuable biomarkers and prognostic indicators have identified in NSCLC. Previous studies showed that lncRNA has an important regulating role in the tumor progression, which can be used as a more efficient as the diagnostic or prognostic molecular biomarkers of tumor therapy [26, 27]. Given the fact that thousands of lncRNAs have been annotated, but the functional and molecular mechanisms has been ambiguity.

In the present study, we revealed signatures of a number of lncRNAs that are aberrantly expressed in human NSCLC tissues, compared to nontumor tissues. We identified a new lncRNA transcript (linc00630), which was significantly upregulated in NSCLC tissues from 90 paired of NSCLC patients. We determined that the high expression level of linc00630 was significantly associated with tumor size, TNM tumor stage, Lymph node status positive and cancer-related death. Patients with higher linc00630 expression exhibited poorer overall survival (OS), indicating that linc00630 expression could serve as a promising prognostic indicator and potential oncogene for HCC patients.

By performed biogical function assays *in vitro* and *in vivo*. Our results showed overexpression of linc00630 could increase the invasion, proliferation and metastasis of NSCLC cells. In order to explore the mechanism of linc00630, we performed RNA pulldown assay and RNA-seq assay with GSEA analysis. Results revealed linc00630 can specified binding with HDAC1 and DDX23 in NSCLC cells. Histone deacetylases (HDACs) remove acetyl groups from histones, resulting in chromatin compaction and decreased accessibility to DNA for interacting molecules such as transcription factors, resulting in compaction of chromatin structure and transcriptional repression [28, 29]. HDACs operate by direct association with DNA-binding factors and by incorporation into large multifunctional repressor complexes such as Sin3, NuRD, and PRC2 [30]. As the famous epigenetic modifying factor, have involved in many human disease also including cancer. Our study founding HDAC1 also binding with long non-coding RNAs, and linc00630 could stabilized the protein level of HDAC1 made its have a higher state in NSCLC to exert its oncogenic functions. Besides, DDX23, a member of the DEAD box protein family. DEAD box proteins, characterized by the conserved motif Asp-Glu-Ala-Asp (DEAD), are putative RNA helicases [31, 32]. They are implicated in a number of cellular processes involving alteration of RNA secondary structure, such as translation initiation, nuclear and mitochondrial splicing, and ribosome and spliceosome assembly [33]. Based on their distribution patterns, some members of this family are believed to be involved in embryogenesis, spermatogenesis, and cellular growth and division [34, 35]. And in our study, we demonstrated DDX23 could initiation the translation of linc00630 by physical binding with the promoter of linc00630. DDX23 made linc00630 has a higher level in NSCLC to exert its oncogenic functions. Results form RNA-seq data showed the Notch signaling pathway which occupies the top one place is a highly conserved cell signaling pathway present in most multicellular organisms and have been demonstrated that promotes proliferative signaling during neurogenesis [36, 37]. Notch signaling is also dysregulated in many cancers [38, 39]. DDX23-Linc00630-HDAC1 axis is a cascade amplification to Notch signaling pathway to excert its oncogenic regulation.

Since the recent reports, lncRNA might be an important supplement to proteins and other effectors in complex regulatory networks of human cancers. In conclusion, we characterized the lncRNA linc00630 as a novel tumor oncogene in NSCLC. Linc00630 can physically interact with HDAC1 and DDX23 and stabilized the protein level of HDAC1 and regulated by DDX23 at the transcriptional level in NSCLC cells. Linc00630 exerts its carcinogenic activity through activation of the Notch signaling pathway in NSCLC. Linc00630 may serve as prognostic predictor for patients with NSCLC, and the DDX23-Linc00630-HDAC1 axis is a potential therapeutic target for NSCLC treatment. And we open avenues for the use of lncRNAs in identification and treatment of novel diagnostic or predictive biomarkers and targets of NSCLC.

## MATERIALS AND METHODS

### Human tissues

A total of 90 lung cancer specimens and their corresponding adjacent non-tumorous tissues used in our study were obtained from the First Affiliated Hospital of Medical School of Zhengzhou University; Liver cancer specimens from 50 patients and their corresponding adjacent non-tumorous tissues used to analyze linc00630 RNA levels and immune-histochemical sections were collected from the surgical specimen archives of the Cancer Institute of Zhoukou Center Hospital. All human materials were obtained with informed consent and the study was approved by the Ethical Review Committee of the World Health Organization of the Collaborating Center for Research in Human Production, authorized by the Henan Municipal Government.

### Cell culture

The NSCLC cell lines A549, SK-MES-1, 95D, H157, H1299, H1975 and H460, Normal NSCLC cell line HBE were purchased from the American Type Culture Collection and cultured in RPMI 1640 supplemented with 10% fetal bovine serum (Invitrogen, USA). HEK293T was cultured in DMEM supplemented with 10% fetal bovine serum.

### Oligos and vectors

A549 and H1975 cells were planted in six-well plate 24 h before transfection. When they were about 70% confluent, cells were transfected with siRNA targeting specific genes or negative control (RealGene, Nanjing, China) by using the Lipofectamine RNAimax reagent (Invitrogen, USA) according to the protocol provided by the manufacturer. The siRNA sequences for linc00630 were 5′-CAGAAGGAGUGAACUGCUAAG-3′ (Sense) and 5′-UAGCAGUAGACUGGUUCUGGG-3′ (Antisense) for 204 site and 5′-GCAAGCCUGCAUGGUACAUTT -3′ (Sense) and 5′-AUGUACCAUGCAGG CUUGCTT -3′ (Antisense) for 1021 site. pCDNA3.1 plasmid was from Addgene (Cambridge, USA). The pCDNA3.1-Puro vector for LINC00630 overexpression was from Applied Biological Materials (ABM) Incorporation. The transfection reagent PowerFect™ *In Vitro* siRNA was from SignaGen Laboratories (Rockville, USA). All experiments were performed after 48 h of transfection. NSCLC cells tranfected with Vector or p-linc00630 plasmid were screened by puromycin (4 μg/ml) for higher expression efficiency.

### qRT-PCR analysis

Purified total RNA extracted from cell lysates was treated with RNase-free DNase (Roche, Basel, Switzerland) to eliminate possible DNA contamination. Quantitative reverse transcription-PCR (qRT-PCR) was performed with a first-strand cDNA synthesis kit and Maxima SYBR green quantitative PCR master mix (Thermo Fisher Scientific, Waltham, MA, USA) according to the standard protocol described in the instructions accompanying the kit. Quantitative analysis of the data was performed using the Prism 7900 analysis software program (Applied Biosystems, Life Technologies, Carlsbad, CA). Primers are provided in Supplementary Materials. The results were normalized to the level of total GAPDH mRNA detected in each RNA sample. The relative fold changes in gene expression were calculated by the comparative CT method, where CT is the threshold cycle. The results presented are from three independent experiments, and statistical significance was determined with an unpaired two-tailed Student's *t* test.

### Cell proliferation assay

Cell proliferation was assayed by Cell Counting Kit-8 (CCK8) assay (Promega). The transfected cells were plated in 96-well plates (4000 cells per well) 24 h after transfection and cultured at 37°C and 5% CO_2_ atmosphere. CCK8 assay was used to detect the relative cell growth every 24 h according to the instructions of manufacturer. Simply, 20 μl of CCK8 solution was added to each well, and each well was measured spectrophotometrically at 450 nm after incubating for 2 h.

### Cell migration and invasion assays

For migration assay, transfected cells (3 × 10^5^) were plated in the upper chamber of transwell assay inserts (8 mm pores, Millipore, Billerica, MA) containing 200 μl of serum-free 1640 medium. The lower chambers were filled with 1640 containing 10% FBS. After 24 h of incubation, the cells on the filter surface were fixed with methanol, stained with crystal violet, and photographed. Migration was assessed by counting the number of stained cell nuclei from 5 random fields per filter in each group.

### 5′ and 3′ rapid amplification of cDNA ends (RACE) analysis, Subcellular fractionation analysis and assessment of protein-coding potential

RACE analysis and subcellular fractionation analysis were performed as described previously. We determined the protein-coding potential of transcript using an *in vitro* translation assay and a combination of protein-coding potential assessment software.

### Northern blot

Northern blot was performed with 10-μg of purified poly(A) RNAs. RNA were resolved by denaturing agarose gel electrophoresis (Ambion, USA), and transferred to Hybond-XL membranes (GE Healthcare, USA). LncRNAs were detected using DIG-labeled probes.

### *In Situ* hybridiation (ISH)

The *in situ* detection of linc00630 was performed on 6-μm formalin-fixed, paraffin-embedded sections using DIG-labeled miRCURYTM detection probe (Exiqon, Denmark). Positive controls and scrambled control RNAs were included for each hybridization procedure and analyzed using a Nikon 80i mociroscope with Nikon NIS-elemetns F2.3 software (Nikon, Japan).

### Luciferase assay

NSCLC cells were transfected with the plasmids expressing the designated combinations of p-linc00630 and other relevant siRNAs at 1.0 μg and 100 ng of Renilla luciferase with lipofectamine 2000 (invitrogene, USA). 24 hours after transfection, the cells were collected to detected luciferase activity using the dual-luciferase reporter assay system (Promega, USA). Luciferase activity was measured by using a BD-Monolight 3010 luminometer (BD biosciences, USA). Transfection efficiency was normalized by dividing the luciferase activity of the construct to the corresponding Renilla luciferase activity.

### RNA pull-down assay, mass spectrometry and RNA immunoprecipitation (RIP)

The experiments were performed as described [40]. RNA immunoprecipitation (RIP) RIP experiments were performed using a Magna RIP RNA-Binding Protein Immunoprecipitation Kit (Millipore) according to the manufacturer's instructions. Antibodies of HDAC1 and DDX23 were from Santa Cruz Biotechnology.

### Sequence alignment and transcriptome Assembly

The University of California Santa Cruz (UCSC) Genome Browser (http://genome.ucsc.edu) is an online public tool providing access to a growing database of genomic sequence and annotations of various organisms for visualization, comparison and analysis.

### Xenograft experiment

Lentivirus packaging and production of p-linc00630 was performed in A549 cells or Vector infection A549 cells were collected and injected into either side of the posterior flank of the same male BALB/c nude mouse. The tumor volumes and weights were measured every 2 days in the mice; the tumor volumes were measured as length × width2 × 0.5. 30 days after injection, the mice were sacrificed, the tumor weights were measured, and the tumors were collected for further analysis. The linc00630 levels were determined by qRT-PCR. All procedures were approved by the Animal Ethic Review Committee of Henan Medical Experimental Animal Care Commission.

### Immunohistochemistry

Human lung tumor tissue sections were immunostained for HDAC1 and DDX23. Antibodies was from Santa Cruz Biotechnology.

### Statistical analysis

The ANOVA test was used to assess the significance of the differences among the experimental groups. The results are represented as means ± standard deviation (S.D.). Results were presented as the means ± standard deviation (SD) or Standard Error of Mean (SEM) of 3 separate assays. Statistical differences were determined by a two-tailed *t* test. Cumulative survival was evaluated using the Kaplan-Meier method, and differences were assessed using the log-rank test. A Kaplan–Meier survival curve was drawn using Graphpad Prism 7 (Graphpad Software Company, USA), and the significance was calculated with the log-rank value. A *P* value < 0.05 was considered to indicate statistical significance.

## SUPPLEMENTARY FIGURES AND TABLE


