# Supplementary material for: DDX23-Linc00630-HDAC1 axis activates the Notch pathway to promote metastasis
Source: Oncotarget. 2017 Apr 17;8(24):38937–49. doi: 10.18632/oncotarget.17156 (PMC5503584; doi:10.18632/oncotarget.17156)
Supplement: Supplementary file 1 [file oncotarget-08-38937-s001.pdf]

DDX23-Linc00630-HDAC1 axis activates the Notch pathway to promote metastasis

Supplementary Materials

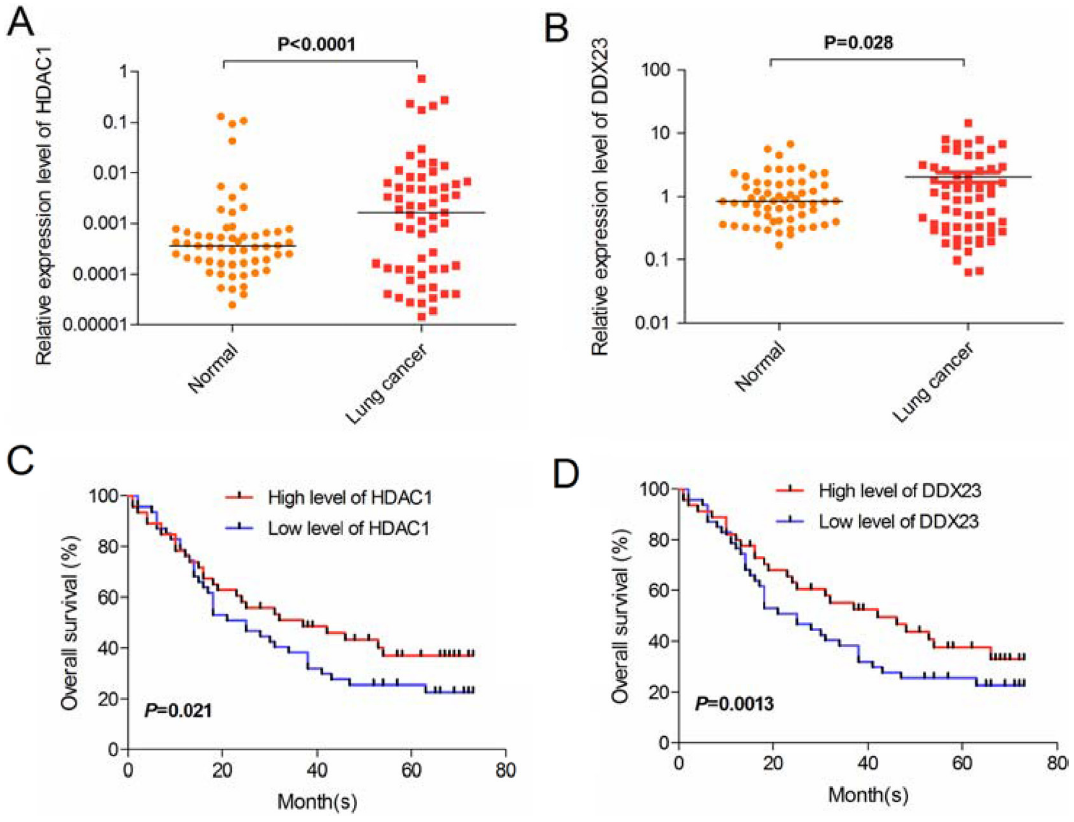

Supplementary Figure 1: HDAC1 and DDX23 are overexpressed and all predicted the overall survival of NSCLC. (**A**) HDAC1 was significantly overexpressed in NSCLC tissues. (**B**) DDX-23 was significantly overexpressed in NSCLC tissues. (**C**) High level of HDAC1 predicted the worse survival of NSCLC patients. (**D**) High level of DDX-23 predicted the worse survival of NSCLC patients.

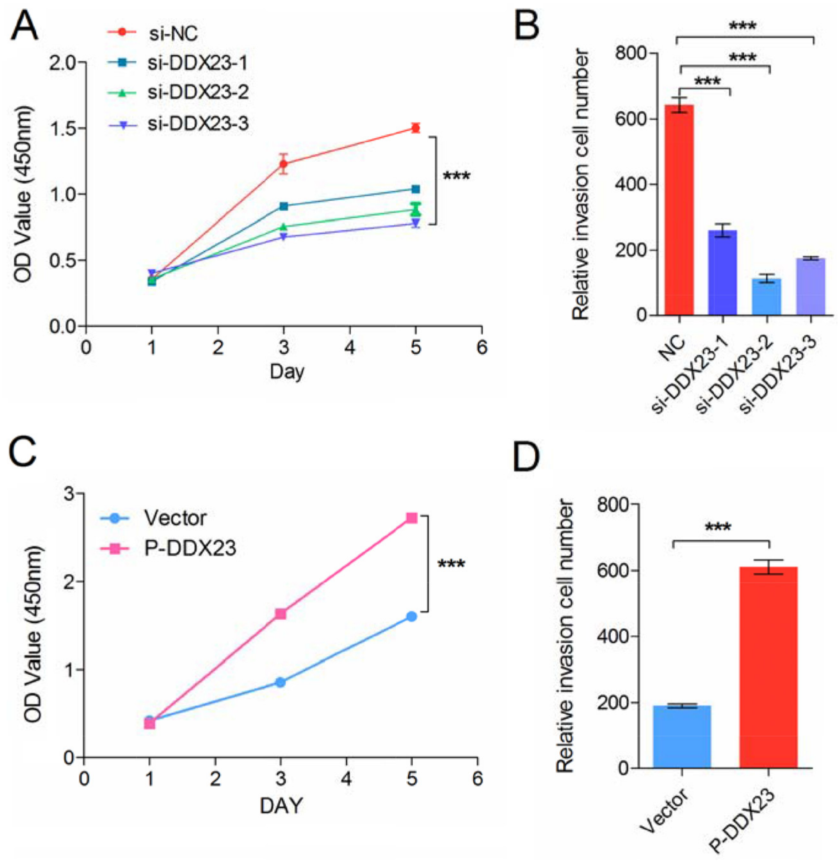

Supplementary Figure 2: DDX-23 could promote proliferation and invasion of NSCLC. (**A**) The growth rate of NSCLC cells when knockdown of linc00630. (**B**) The invasive ability of NSCLC cells when knockdown of linc00630. (**C**) The growth rate of NSCLC cells when overexpression of linc00630. (**D**) The invasive ability of NSCLC cells when overexpression of linc00630.

Supplementary Table 1: Relationship between linc00630expression and clinicopathologic factors of patients with Lung cancer

Parameter	No. of patients	Linc00630 (low)	Linc00630 (high)	*P*-value
Sex				0.4506
male	70	30	40	
female	20	12	18	
Age (yr)				0.1223
< 60	60	27	33	
≥ 60	30	17	13	
Tumor differentiation				0.0321
I	10	3	7	
II	60	10	50	
III	20	5	15	
Tumor size (cm)				0.0061
≤ 5	50	30	20	
> 5	40	22	28	
Differentiation grade				0.212
Well-moderate	54	23	31	
Poor-undifferentiation	36	12	24	
T stage				0.0013
T1–T3	48	18	30	
T4	42	31	11	
Lymph node status				
Negative	40	10	30	0.0071
Positive	50	20	30	
Distant metastasis				0.2107
M0	48	30	18	
M1	42	22	20	
TNM stage				< 0.0001
I–II	47	27	20	
III–IV	43	21	22	
Lymphatic invasion				0.007
Negative	20	3	17	
Positive	70	19	51	
Venous invasion				0.0811
Negative	44	22	22	
Positive	46	26	20	
